# Peripheral primitive neuroectodermal tumor of the kidney in a 51-year-old female following breast cancer: A case report and review of the literature

**DOI:** 10.3892/ol.2014.2695

**Published:** 2014-11-10

**Authors:** JINJING ZHONG, NI CHEN, XUEQIN CHEN, JING GONG, LING NIE, MIAO XU, QIAO ZHOU

**Affiliations:** Department of Pathology, West China Hospital of Sichuan University, Chengdu, Sichuan 610041, P.R. China

**Keywords:** renal peripheral primitive neuroectodermal tumor, breast adenocarcinoma, immunohistochemistry, fluorescent *in situ* hybridization, Ewing sarcoma breakpoint region 1, adjuvant therapy

## Abstract

Peripheral primitive neuroectodermal tumor/Ewing’s sarcoma (pPNET/EWS) is an aggressive type of sarcoma that is rarely observed in the kidney. pPNET of the kidney principally occurs in young patients (<50 years old) and is very rare in older patients (≥50 years old). Additionally, only six cases of pPNET of the kidney have been reported in the literature in older patients (≥50 years old), and pPNET as a secondary primary tumor has rarely been reported. The current study presents a case of renal pPNET in a 51-year-old female who had been surgically treated for breast carcinoma and administered with adjuvant chemotherapy five years prior to hospitalization for pPNET. A computed tomography scan identified a tumor in the lower pole of the right kidney, which was treated by nephrectomy. Immunohistochemistry demonstrated diffuse, strong membranous positivity for cluster of differentiation (CD)99, positive nuclear staining for friend leukemia integration 1, and negative staining for Wilms’ tumor 1 and other markers. Fluorescence *in situ* hybridization (FISH) analysis of the EWS breakpoint region 1 (EWSR1) demonstrated the characteristic EWSR1 translocation. The patient declined chemotherapy or radiotherapy but accepted traditional Chinese medicine. No evidence of recurrence was observed eight months after diagnosis. Only two cases of renal pPNET with a history of an earlier or synchronous primary cancer were reported in the literature from the USA and Germany, respectively. To the best of our knowledge, the present case is the first FISH-confirmed renal pPNET in an older patient following breast adenocarcinoma.

## Introduction

Peripheral primitive neuroectodermal tumor/Ewing’s sarcoma (pPNET/EWS) is an aggressive type of sarcoma characterized by the t(11; 22)(q24;q12) translocation and overexpression of the cluster of differentiation (CD)99/MIC2 gene (1). pPNET is predominantly considered to be a malignant bone or soft tissue tumor of children and young adults that is uncommon in individuals aged >30 years (2). pPNET of the kidney is very rare, and is even rarer in older patients or in patients who have received treatment for a different type of cancer. The current study presents the first known case of renal pPNET following breast adenocarcinoma in a 51-year-old female. Written informed consent was obtained from the patient’s family.

## Case report

### Patient presentation

**Figure 1:**
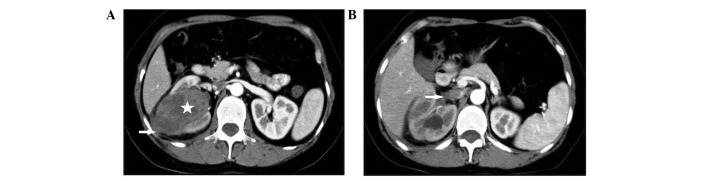
(A) Enhanced computed tomography (CT) image demonstrating a large mass replacing the lower part of the right kidney (star); the mass had invaded the perirenal space and Gerota’s fascia (arrow). (B) Enhanced CT image of the upper abdomen reveals a thrombus in the renal vein and inferior vena cava (arrow).

In December 2013, a 51-year-old female complaining of right flank pain was admitted to the West China Hospital of Sichuan University (Chengdu, China). Urine examination determined positive occult blood; however, all other laboratory analyses were unremarkable. Ultrasonography (USG) and computerized tomography (CT) scans revealed a 7×4-cm mass with an irregular contour in the lower pole of the right kidney, which involved the renal vein and the inferior vena cava (IVC). Enhanced CT scans identified extension of the tumor into the perirenal space and Gerota’s fascia (Fig. 1). The patient had been treated by radical mastectomy and adjuvant chemotherapy for a left breast adenocarcinoma five years prior to the current hospitalization. No evidence of breast cancer recurrence was observed.

### Pathological observations

**Figure 2:**
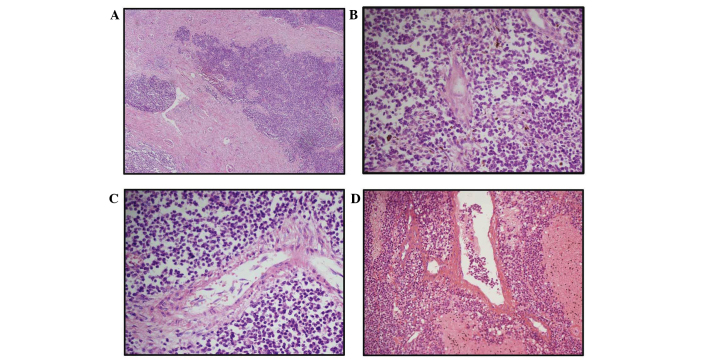
(A) Histological examination of the kidney demonstrates that the tumor mass had infiltrated the cortex and medulla (original magnification, ×40). (B) Small, round tumor cells with scanty cytoplasm and round nuclei (original magnification, ×400). (C) Neoplastic cells infiltrating blood vessels (original magnification, ×400). (D) Similar neoplastic cells in the tumor thrombus (original magnification ×400). Staining, hematoxylin and eosin.

The patient received radical nephrectomy with resection of tumor thrombi in the IVC and right renal vein. Grossly, the kidney measured 11.5×8.5×4.6 cm and protruded from the kidney surface. On cut surface, the nodular, solid tumor measured 7.0×4.5×3.5 cm, with areas of hemorrhage and necrosis, as well as infiltration into the medulla and cortex. Analysis of hematoxylin and eosin-stained sections revealed that the tumor was composed of monotonous, small, blue round cells, two to three times the size of lymphocytes, with hyperchromatic nuclei and scanty cytoplasm. Furthermore, vague rosette-like patterns or perivascular concentrations of tumor cells were observed (Fig. 2).

**Figure 3:**
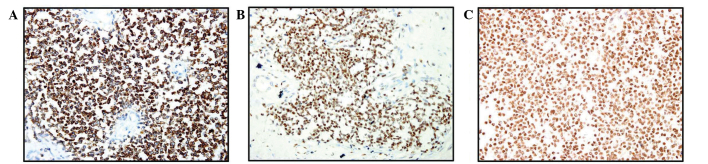
Immunohistochemical staining reveals that the tumor cells were positive for (A) cluster of differentiation 99, (B) friend leukemia integration 1 and (C) integrase interactor-1 (original magnification, ×400).

An immunohistochemical panel demonstrated strong, diffuse membranous positivity for CD99 and nuclear staining for friend leukemia integration 1 (FLI1) in the tumor sections (Fig. 3A and B, respectively). No loss of integrase interactor-1 (INI-1) protein expression levels were observed, as determined by the nuclear positivity for INI-1 (Fig. 3C), and the Ki-67 nuclear labeling index was ~10%. Additionally, the tumor cells were negative for desmin, myogenin, leukocyte common antigen (LCA), CD20, CD3, cytokeratin, P63 and Wilms’ tumor 1 (WT-1).

**Figure 4:**
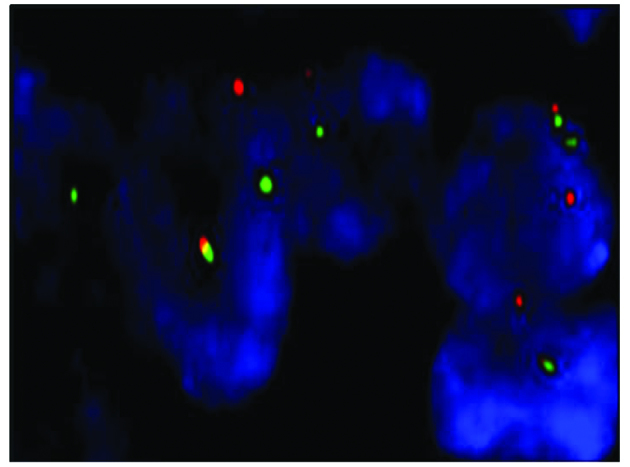
Fluorescence *in situ* hybridization analysis using a Vysis LSI EWSR1 Dual Color, Break Apart Rearrangement probe for 22q12 demonstrates the green and red probe breaking apart, confirming the Ewing’s sarcoma breakpoint region 1 translocation.

Fluorescence *in situ* hybridization (FISH) analysis using a Vysis LSI EWSR1 (22q12) Dual Color, Break Apart Rearrangement probe (Abbott Molecular, Inc., Des Plaines, IL, USA) was performed and revealed the characteristic EWSR1 translocation, confirming the diagnosis of pPNET of the kidney (Fig. 4).

### Follow-up

The patient declined chemotherapy following surgery but accepted traditional Chinese medicine (six ingredient rehmannia pill). The patient has been followed up for the previous eight months with scheduled CT and USG scans, and periodic tumor marker monitoring. No evidence of recurrence or metastasis has been observed thus far.

## Discussion

pPNET is a small round-cell tumor that predominantly occurs in bone and soft tissue; however, pPNET is occasionally reported in visceral organs, such as urogenital, intra-abdominal and intrathoracic organs. pPNET of the kidney is uncommon (3–5) and was first reported in 1975 (6). Since the first report, ~150 cases have been published in the medical literature, with the largest series of 24 cases reported by Zöllner *et al* (7). pPNET of the kidney is an aggressive disease with a high metastatic potential that predominantly occurs in children and young adults (median patient age, 28 years) and exhibits a slight male predominance (male:female ratio, 1.2:1) (8). pPNET of the kidney following or synchronous with a different primary cancer is extremely rare, with only two reported cases (from the USA and Germany, respectively) (9,10). To the best of our knowledge, the present case is unique: A female aged >50 years with a history of breast adenocarcinoma that had been treated with chemotherapy five years prior to the diagnosis of renal pPNET. Only six cases of a patient aged >50 years have previously been reported (9,11–14), the clinicopathologic features of which, together with that of the present case, are summarized in Table I. Whether the renal pPNETs developed independently or were associated with the previous cancer is unknown and future studies are required to clarify the issue.

Diagnosis of pPNET is based on morphologic, immunohistochemical and genetic analyses. For example, pPNET of the kidney is characterized by small, round cells with hyperchromatic nuclei, scant to moderate cytoplasms and occasional rosette-like structures (15). However, pPNET of the kidney must still be differentiated from other small round-cell tumors, such as blastemal Wilms’ tumor, malignant lymphoma, small cell carcinoma, rhabdomyosarcoma, poorly differentiated synovial sarcoma and desmoplastic small round-cell tumors. Strong membranous positivity for CD99 and nuclear staining of FLI1 are characteristic features of pPNET; however, they are not definitive features, as CD99 positivity is observed in ~99% of cases (8). Therefore, an immunohistochemical panel consisting CD99, FLI1 and other relevant differential markers is recommended (16). Blastemal Wilms’ tumor is typically observed in patients of a young age (<5 years) and is usually immunohistochemically positive for WT-1 to various extents. Additionally, pPNET can be differentiated from rhabdomyosarcoma, which is positive for desmin and myogenin, and lymphoma, which may be excluded by immunostaining for LCA and other lymphohematopoietic markers (16).

Genetically, pPNET of the kidney is characterized by the chromosomal translocation t(11; 22)(q24;q12), resulting in the production of the EWS/FLI1 fusion gene (17). The EWS gene on chromosome 22 encodes an RNA-binding protein that is disrupted by the t(11;22)(q24;q12) translocation. The FLI1 gene on 11q24 was the first EWS translocation partner identified in the EWS/PNET family of tumors; it is a member of a large family of DNA-binding transcription factors that have a highly conserved 85-amino acid domain, termed the erythroblastosis virus-transforming sequence (ETS) domain (18). The resultant oncogenic fusion gene includes the N-terminal transactivation domain of EWS and the C-terminal DNA activation domain of FLI1, which acts as a powerful transcriptional activator (16). Furthermore, a recent study identified an association between CD99 overexpression and EWS/FLI1 fusion: EWS/FLI1 may downregulate microRNAs (miR), such as miR-30-5p, which may interact with the 3′-untranslated region of CD99 and post-transcriptionally downregulate its expression (19). Additionally, alternative chromosomal translocations with analogous fusion of EWS to other partners have been reported; for example, the rare t(16;21)(p11;q22) translocation and fusion of the ETS-related gene ETS domain to fused in sarcoma has previously been reported in renal pPNET (20). Thus, FISH with negative EWS-FLI1 fusion only cannot completely exclude the diagnosis of pPNET of the kidney (20,21).

pPNET of the kidney is an aggressive tumor with a poor prognosis; a previous study identified that 25–50% of patients presented with metastases and the five-year disease-free survival rate was ~45–55% (22). A review of 116 reported cases of renal pPNET demonstrated consistent results; 33% of patients exhibited metastases at diagnosis and 40% of patients developed metastases following surgery for the primary tumor (8). Treatment strategies for renal pPNET are similar to those for Ewing’s sarcoma, requiring systemic chemotherapy in conjunction with surgery or radiotherapy or both modalities for local tumor control. The benefit of radiotherapy is not clear, however, it may be preferable in locally advanced disease or in cases with Gerota’s fascia involvement (23). Since the cases of patients aged >50 years are rare, no consensus of a recommended treatment strategy has been documented.

In conclusion, although pPNET of the kidney typically occurs in young adults, it has also been reported to occur in older patients. Immunohistochemistry for CD99, FLI1 and other differential markers, as well as FISH analysis for EWSR1 fusion are key in the diagnosis of renal pPNET, which should be included in the differential diagnosis of small-cell kidney tumors in all age groups.

## Figures and Tables

**Table I:** Clinical and follow-up data of cases of renal peripheral primitive neuroectodermal tumor in older patients.

Case report (reference number)	Age, years	Gender	Tumor size, cm	Position	CD99 IHC	EWSR1 FISH	Treatment strategy	Chemotherapeutic agents	Recurrence or metastasis	Follow-up period	Outcome at time of report
Tariq *et al* (11)	57	Female	UNK	UNK	+	+	Nephrectomy and multimodal treatment	UNK	Pulmonary metastasis at diagnosis	8 years	Alive with stabilized lung metastasis
Mandal *et al* (12)	50	Female	16.0	L	+	NC	Nephrectomy and chemotherapy	Vincristine, mesna, ifosfamide and doxorubicin	Retroperitoneal lymph nodal metastasis at diagnosis	1 year	Alive
Wedde *et al* (13)	73	Male	UNK	R	+	+	Nephrectomy and chemotherapy	UNK	No	>7 months	Alive
Koski *et al* (14)	78	Female	10.9	L	+	NC	Nephrectomy	NC	Pulmonary metastasis without chemotherapy	2 weeks	Deceased
Jimenez *et al* (9)	69	Female	5.0	R	+	NC	Nephrectomy and chemotherapy	Carboplatinum, VP-16, taxol interferon and estramustine etoposide	Lung and bone metastasis six months after chemotherapy	25 months	Deceased
Jimenez *et al* (9)	50	Male	UNK	UNK	+	NC	Nephrectomy	NC	UNK	UNK	UNK
Present case	51	Female	11.5	R	+	+	Nephrectomy	NC	No	8 months	Alive

CD, cluster of differentiation; IHC, immunohistochemistry; EWSR1, Ewing’s sarcoma breakpoint region 1; FISH, fluorescent *in situ* hybridization; UNK, unknown; L, left kidney; R, right kidney; NC, not conducted.

## Abbreviations

pPNET: peripheral primitive neuroectodermal tumor
ES: Ewing’s sarcoma
USG: ultrasonography
CT: computerized tomography
FISH: fluorescent *in situ* hybridization
IHC: immunohistochemistry
